# A model study for causal relationships between voltage and calcium dynamics

**DOI:** 10.1186/1471-2202-12-S1-P359

**Published:** 2011-07-18

**Authors:** Pablo Chamorro, Daniele Marinazzo, Rafael Levi, Francisco B Rodriguez, Pablo Varona

**Affiliations:** 1 Grupo de Neurocomputación Biológica, Escuela Politécnica Superior, Universidad Autónoma de Madrid, Spain; 2 Lab. of Neurophysiology and New Microscopies, CNRS UMR 8154, Univ. Paris Descartes, France

## 

Intracellular mechanisms directly or indirectly, influence the electrical activity of neurons in different time scales. These subcellular processes play a crucial role in generating transient dynamics and may shape firing patterns of individual cells and circuit activity in the nervous system [1-3]. Slow dynamics can contain a short-term history of a neuron and predispose it to prior activity-dependent (or preferred input-output) responses [4]. The precise temporal distribution of spiking activity can also have as a substrate the slow calcium dynamics [5].

We have addressed this question using conductance based models including a description of calcium dynamics. In order to explore their mutual interaction, we evaluated the strength of the causal relationships between the calcium concentration and the membrane potential with Granger Causality (GC). GC is an efficient way to investigate cause-effect relationships between time series, and is currently the state of the art method for this kind of analysis in neural data. For this study we applied Kernel Granger Causality (KGC), a recently proposed approach which allows a straightforward extension to the nonlinear case [6]. The causality index was evaluated for several parameters of the model, such as the order of regression, the delay and the degree of nonlinearity. Given the oscillatory nature of the signals, we also employed a modified approach which allows evaluating causality between the phases of the oscillations.

The causal relationship between voltage and calcium was analyzed for different activity modes in the models, i.e., regular spiking, regular bursting, irregular spiking and irregular bursting. The results show several evolving asymmetries between the causality in the voltage -> calcium direction and the other way around. These asymmetries can be related to the different temporal structure of the spiking and spiking-bursting regimes.

**Figure 1:**
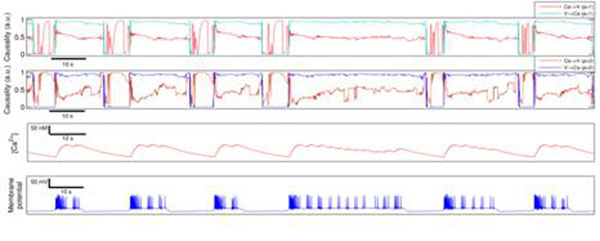
Causality analysis between voltage and calcium for one of the conductance based models evaluated. The first top rows display the causality between the membrane potential and the calcium concentration depicted in the last two rows, respectively. The calcium and voltage time series were approximated by a stationary Markov process of order 2 and the KGC algorithm was applied using a polynomial kernel with p = 1 (first row) and p = 2 (second row).

This study contributes to a better interpretation of the calcium->voltage and voltage->calcium analysis of experimental recordings. In particular, we compare the results of these simulations with the causality analysis performed in simultaneous recordings of membrane potential activity and calcium imaging in CPG neurons from the crab *Carcinus* maenas. Figure 1.
